# Laparoscopic low anterior resection for rectal cancer with rectal prolapse: a case report

**DOI:** 10.1186/s13256-017-1555-1

**Published:** 2018-02-06

**Authors:** Ryusei Yamamoto, Yasuji Mokuno, Hideo Matsubara, Hirokazu Kaneko, Shinsuke Iyomasa

**Affiliations:** Department of Surgery, Yachiyo Hospital, 2-2-7, Sumiyoshi-cho, Anjo-shi, Aichi, 446-8510 Japan

**Keywords:** Rectal cancer, Rectal prolapse, Laparoscopic, Low anterior resection, Prolapsing technique

## Abstract

**Background:**

Rectal cancer with rectal prolapse is rare, described by only a few case reports. Recently, laparoscopic surgery has become standard procedure for either rectal cancer or rectal prolapse. However, the use of laparoscopic low anterior resection for rectal cancer with rectal prolapse has not been reported.

**Case presentation:**

A 63-year-old Japanese woman suffered from rectal prolapse, with a mass and rectal bleeding for 2 years. An examination revealed complete rectal prolapse and the presence of a soft tumor, 7 cm in diameter; the distance from the anal verge to the tumor was 5 cm. Colonoscopy demonstrated a large villous tumor in the lower rectum, which was diagnosed as adenocarcinoma on biopsy. We performed laparoscopic low anterior resection using the prolapsing technique without rectopexy. The distal surgical margin was more than 1.5 cm from the tumor. There were no major perioperative complications. Twelve months after surgery, our patient is doing well with no evidence of recurrence of either the rectal prolapse or the cancer, and she has not suffered from either fecal incontinence or constipation.

**Conclusions:**

Laparoscopic low anterior resection without rectopexy can be an appropriate surgical procedure for rectal cancer with rectal prolapse. The prolapsing technique is useful in selected patients.

## Background

Rectal prolapse is a pelvic disorder that typically occurs in older women with an incidence of 0.25% [1, 2]. Some reports state that laparoscopic surgery for transabdominal rectopexy is recommended because it is less invasive than open surgery [3, 4]. Laparoscopic low anterior resection (Lap-LAR) has been successfully adopted for rectal cancer because of its favorable short-term outcomes [5–9]. However, the coexistence of rectal cancer and rectal prolapse is extremely rare; thus, the etiology and treatment policy is still unclear. To the best of our knowledge, only three cases of rectal cancer with rectal prolapse (RCRP) have been reported [10–12], and none of these patients was treated with laparoscopic surgery. We present a patient with RCRP that was successfully resected using Lap-LAR with a favorable outcome. We also review the relevant literature on this unusual presentation.

## Case presentation

**Fig. 1:**
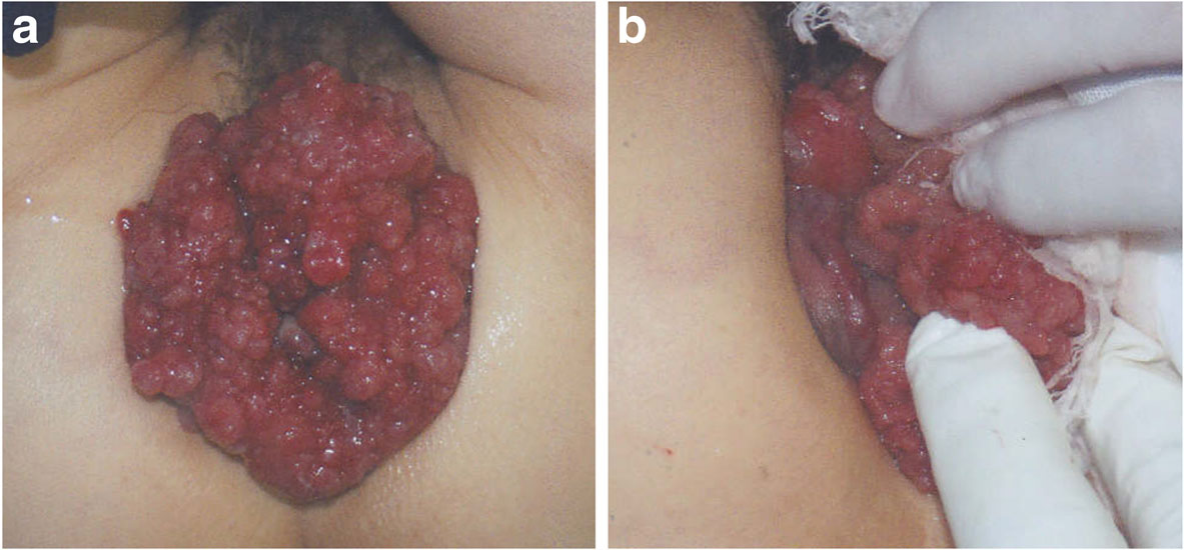
Physical examination showing complete rectal prolapse of the entire thickness of the rectum with a soft, 7-cm tumor with ulceration. **a**: frontal view, **b**: lateral view

**Fig. 2:**
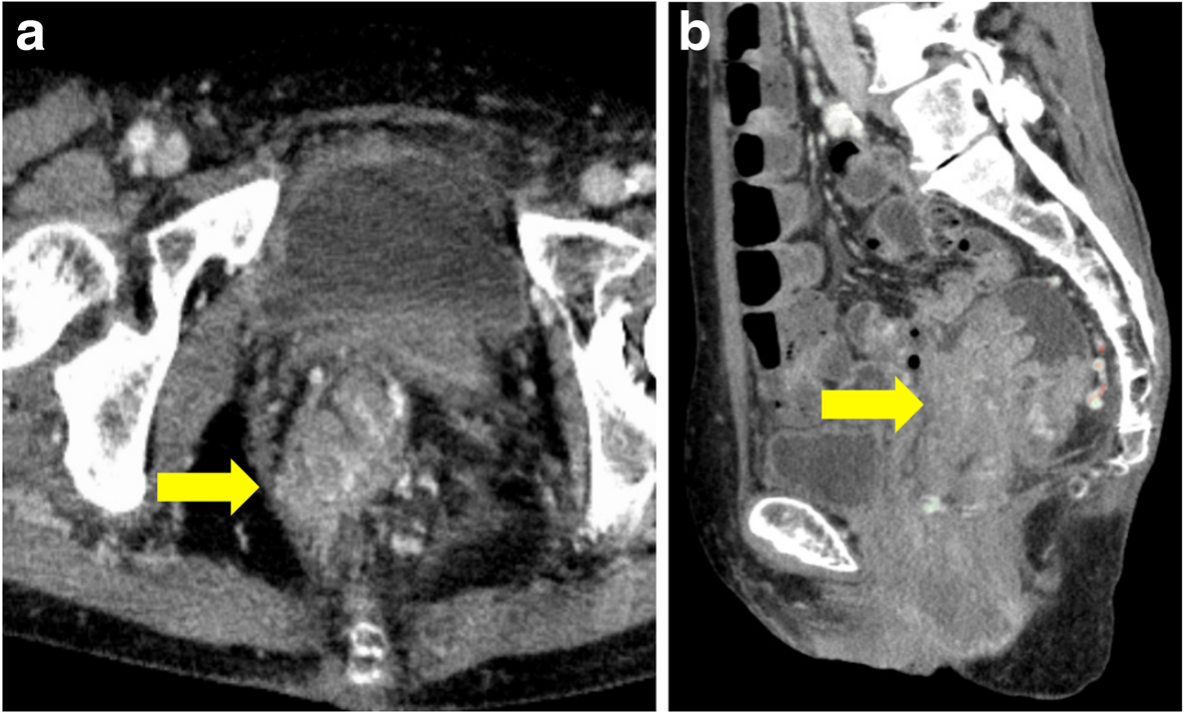
Computed tomography scan showing the lower rectum filled with a tumor measuring 7 × 6 cm, enhanced with contrast medium. The arrows pointing to the tumor. **a** axial plane, **b** sagittal plane

**Fig. 3:**
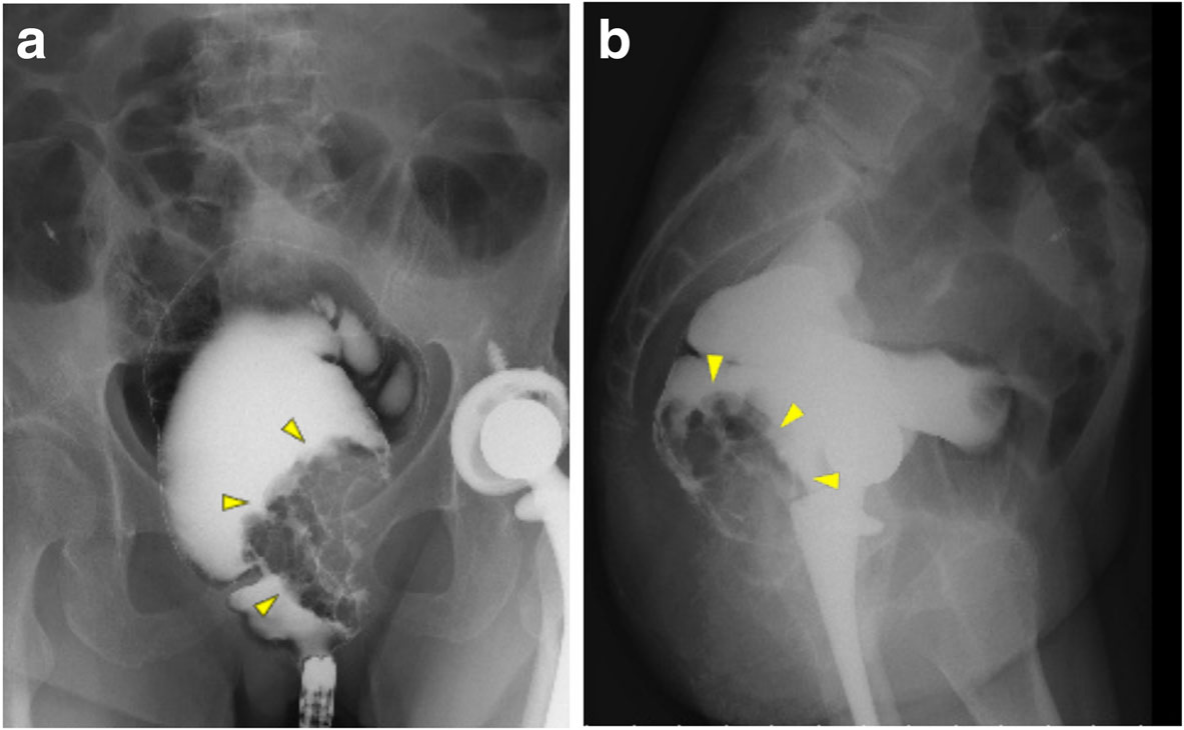
Gastrografin enema showing a tumor located in the posterior wall of the lower rectum. The arrows pointing to the tumor. **a** coronal plane, **b** sagittal plane

**Fig. 4:**
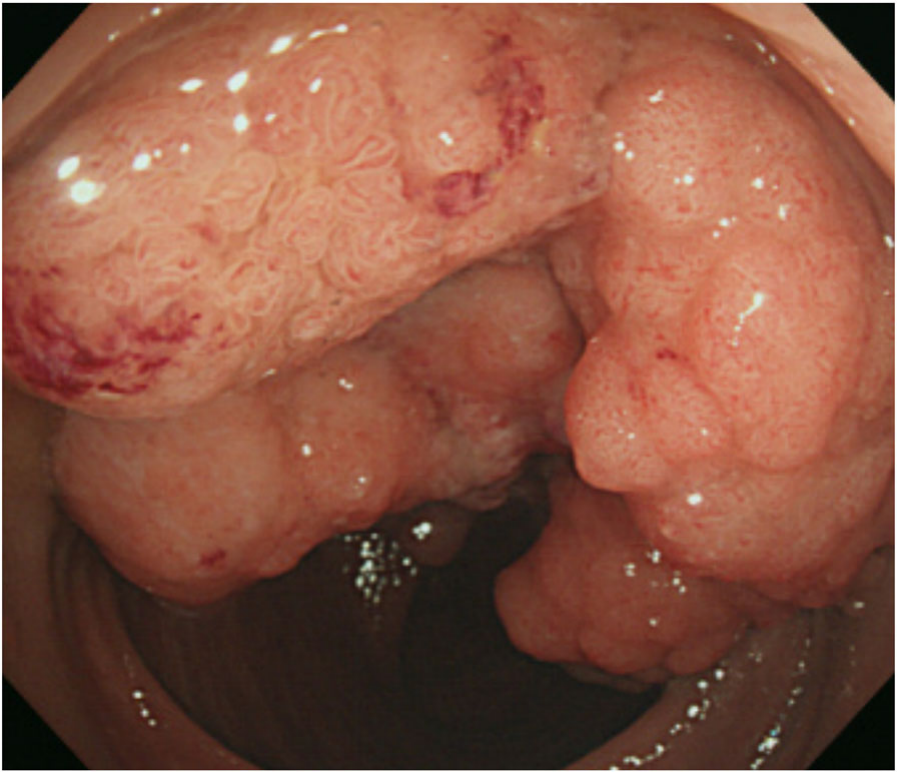
Colonoscopy showing a large villous tumor in the lower rectum

A 63-year-old Japanese woman was referred to our hospital after suffering from rectal prolapse with a mass and rectal bleeding for 2 years. An examination revealed complete rectal prolapse of the entire thickness of the rectum with a soft, ulcerated tumor, 7 cm in diameter, included in the prolapse (Fig. 1). The tumor and prolapse were easily reduced manually; the distance from the anal verge to the tumor was 5 cm. The anal sphincter tone was slightly diminished. The patient’s laboratory data were normal except for anemia (hemoglobin level, 5.9 g/dL). Tumor markers were within normal limits: carcinoembryonic antigen was 2.8 and carbohydrate antigen 19-9 was 6.2. A computed tomography scan revealed that the lower rectum was filled with a tumor measuring 7 × 6 cm, which was enhanced with contrast medium (Fig. 2). There was no evidence of direct invasion of the tumor around the rectum, and no evidence of lymph node involvement or distant metastasis. A gastrografin enema revealed that the tumor was located in the posterior wall of the lower rectum (Fig. 3). Colonoscopy showed a large villous tumor in the lower rectum. The cauliflower shape of the tumor caused the edge to protrude laterally from its base, and the rim of the tumor appeared friable (Fig. 4). Biopsies of the tumor revealed adenocarcinoma. These findings suggested that the large, partially ulcerated tumor was not more than a T1 stage. We defined her cancer as clinical stage 1; cT1, cN0, cM0 according to the Union for International Cancer Control Classification of malignant tumors, 7^th^ edition. Therefore, we did not consider neoadjuvant chemoradiotherapy. Thus, we planned to perform Lap-LAR. To preserve the anal sphincter and to retract the part of the cauliflower-shaped tumor protruding over the wall of the rectum, ensuring a negative distal margin, we also planned to use a prolapsing technique.

**Fig. 5:**
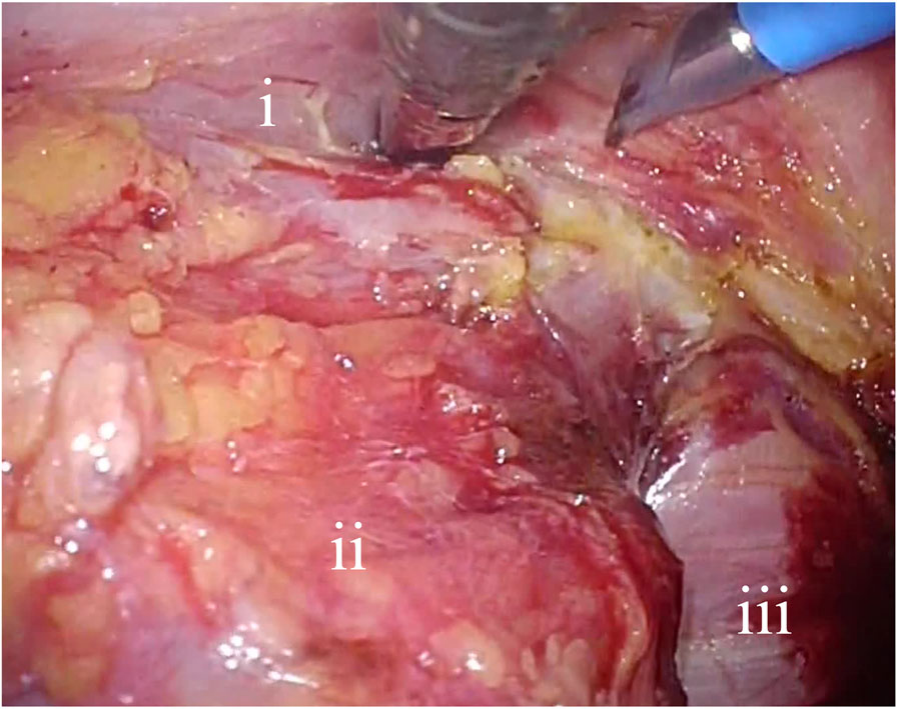
Laparoscopic view showing mobilization of the rectum using the total mesorectal excision technique; the dissection is extended distally to expose the entire circumference of the levator ani muscle. *i* vagina; *ii* rectum; *iii* levator ani muscle

**Fig. 6:**
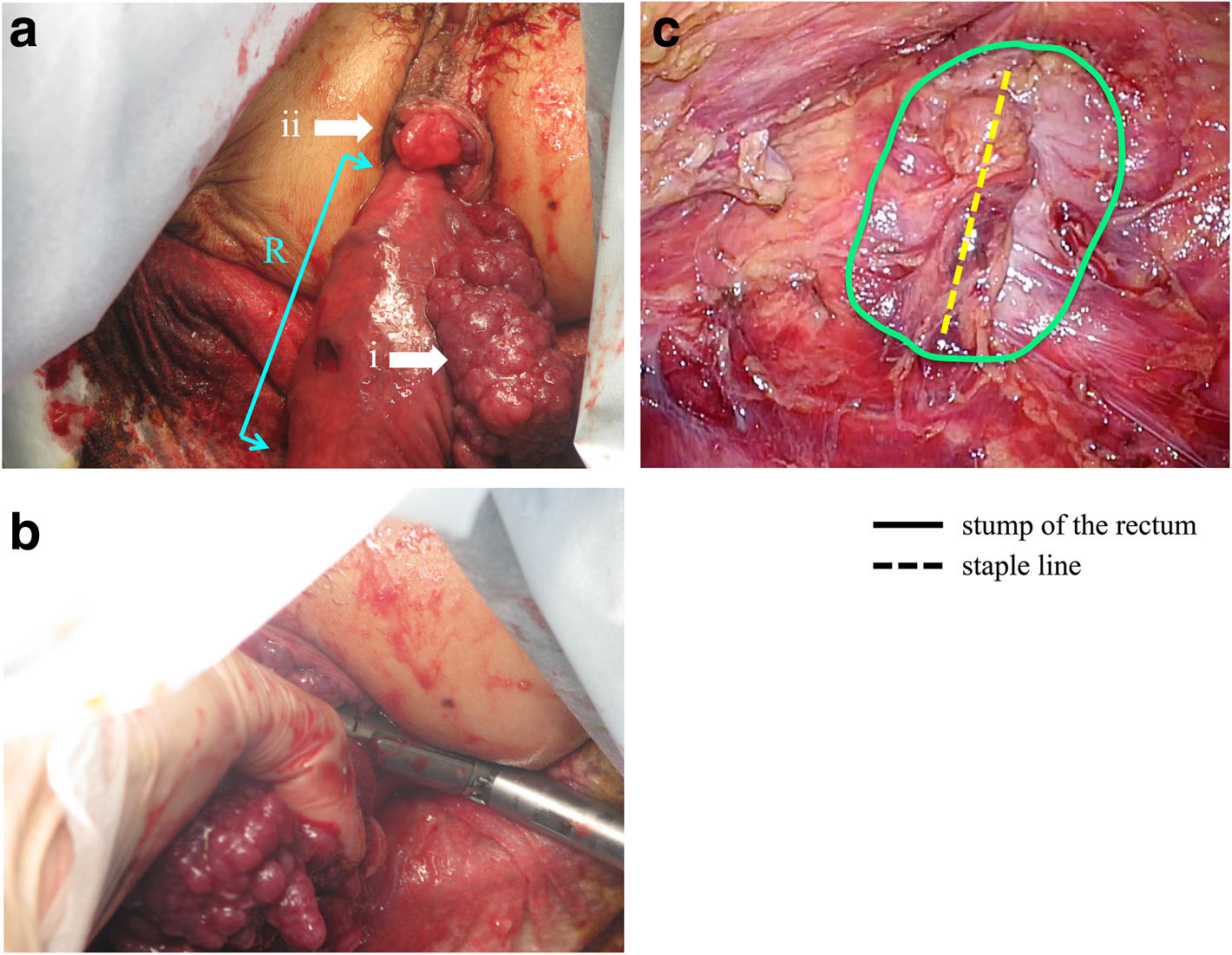
**a** The distal rectum is everted and pulled outside of the anus. *i* tumor; *ii* anus; *R* everted rectum. **b** The distal rectum is transected under direct visualization. **c** Laparoscopic view showing the stump of the rectum reduced into the pelvis after resection

Our patient was placed in the lithotomy position under general anesthesia, and five ports were inserted. We dissected the lymph nodes from the origin of the inferior mesenteric artery. We preserved the left colic artery and ligated and divided the superior rectal artery. The sigmoid colon and the rectum were mobilized using the total mesorectal excision technique, and the dissection was extended distally to expose the entire circumference of the levator ani muscle (Fig. 5). The proximal colon was transected using an endoscopic stapler. Then, we grasped the stump of the rectum using a forceps introduced anally and gently withdrew the stump under laparoscopic assistance. The distal rectum was easily everted and pulled outside of the anus (Fig. 6a). We transected the distal rectum 1.5 cm distal to the lower edge of the lesion under direct visualization, using the same stapler (Fig. 6b). An intraoperative frozen section revealed that the surgical margin was negative for tumor involvement. We reduced the distal rectum, through the anus, back into the pelvis (Fig. 6). Then, we performed a minilaparotomy with a 4-cm incision at the umbilical port site to extract the proximal colon. We resected an extra 10 cm of proximal colon to create the proper length for an anastomosis and attached a 29-mm anvil head. We performed an end-to-end anastomosis using a double-stapling technique with a circular stapler. The operative time was 194 minutes, and the blood loss was 10 mL.

**Fig. 7:**
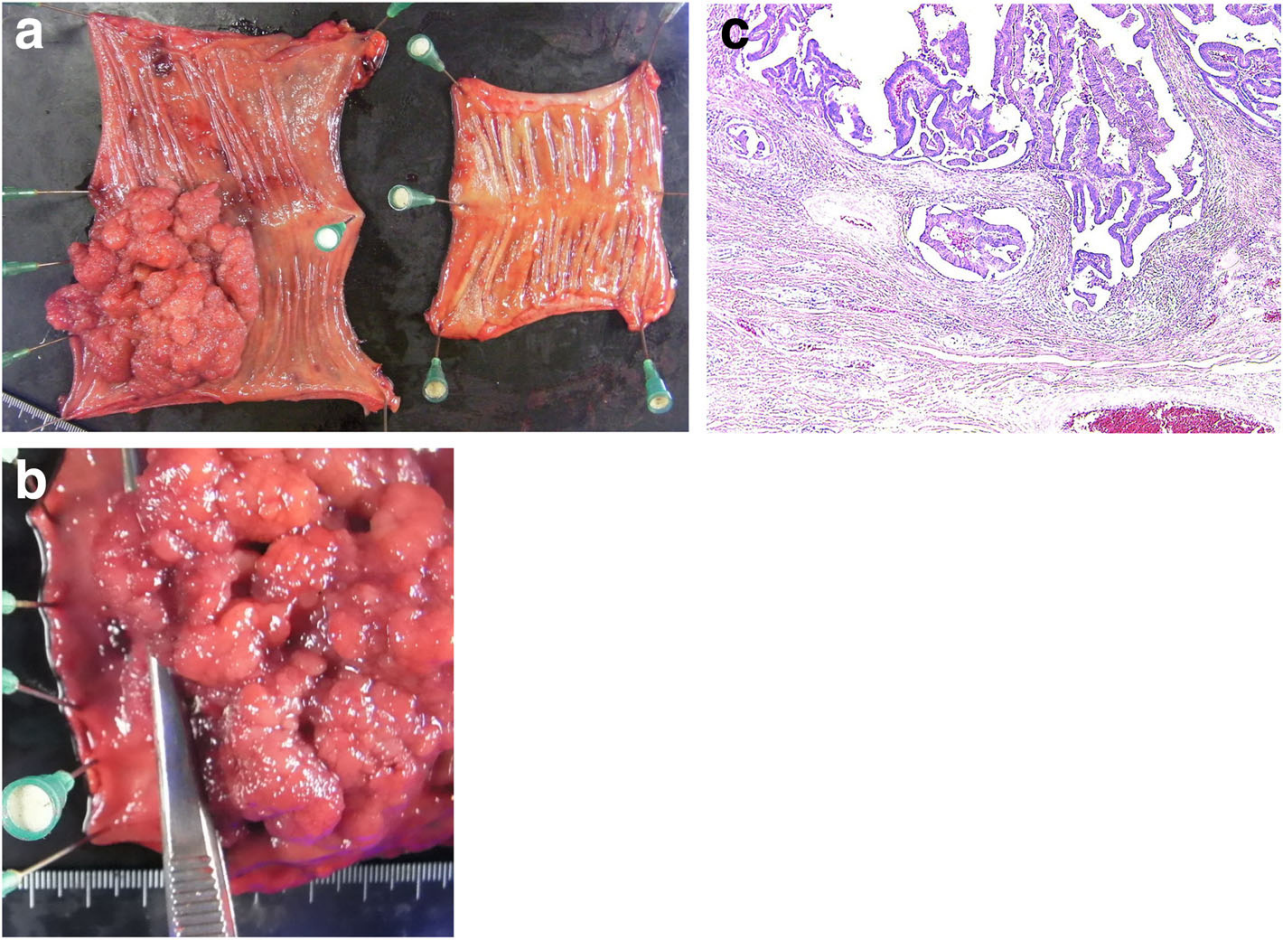
**a** Gross examination showing an ulcerated, cauliflower-shaped, soft tumor, measuring 7 × 8 × 3 cm, located in the lower rectum; the proximal colon is also resected. **b** The distal margin of the rectum is more than 1.5 cm from the tumor. **c** Histologic examination showing a moderately differentiated tubular adenocarcinoma, almost entirely intramucosal, with focal invasion of the submucosal layer

On gross examination of the resected specimen, a cauliflower-shaped soft tumor with an ulcer was located in the lower rectum. The tumor measured 7 × 8 × 3 cm, and the distal margin of the resected rectum was more than 1.5 cm from the tumor. Histological examination of the tumor showed moderately differentiated tubular adenocarcinoma without regional lymph node metastasis. Almost the entire tumor was intramucosal, with focal invasion of the submucosal layer (stage 1; pT1, pN0, pM0) (Fig. 7). Our patient had an uneventful postoperative course. She had no fecal incontinence or constipation. Twelve months after surgery, she is doing well with no evidence of recurrence of either her rectal prolapse or her cancer.

## Discussion

We elucidate here two important clinical issues: Lap-LAR without rectopexy can be an appropriate treatment for RCRP, and the prolapsing technique is useful for these patients.

Our review of the literature found five reports of colorectal cancer resection in patients with rectal prolapse; three of these patients had rectal cancer [10–14] (Table 1). However, only one patient was treated with low anterior resection; the others were treated using a perineal approach, and none was treated using laparoscopy. To the best of our knowledge, ours is the first report of RCRP treated with Lap-LAR. Because of the rarity of this condition, the etiology and treatment policy are still unclear. However, it is evident that priority should be placed on the oncological view, and we should follow the principles of surgical resection for rectal cancer. Laparoscopic surgery for rectal cancer has recently become the standard of care [5–9].

**Table 1:** Patients with rectal prolapse and colorectal cancer

Author	Years	Age (y)	Sex	Location of tumor	Pathology	Procedure	Recurrence of rectal prolapse	Recurrence of cancer
Yamazaki *et al*. [13]	1999	76	Female	Sigmoid colon	Carcinoma	Sigmoidectomy + Rectopexy	No	No
Erikoğlu *et al.* [10]	2004	63	Female	Rectum	Well diff. adenocarcinoma	Low anterior resection	No	No
Karmercan *et al.* [11]	2007	33	Female (pregnant)	Rectum	Moderately diff. adenocarcinoma	Altemeier + Proctocolectomy	No	No
Bounovas *et al.* [14]	2007	85	Female	Sigmoid colon	Adenocarcinoma	Sigmoidectomy + Rectopexy	No	No
Nabi [12]	2015	77	Female	Rectum (After Hartmann)	Adenocarcinoma	Intersphincteric perineal proctectomy	No	No

*diff* differentiated

For rectal prolapse, there are two approaches to surgical repair: transabdominal and perineal. Many reports on the surgical treatment of rectal prolapse show that recurrence rates after abdominal repair are lower than after perineal repair [15–25]. Concomitant sigmoid resection is recommended for patients who have preexisting constipation [4, 26]. In a low anterior resection, a large part of the sigmoid colon is removed, and the reconstructed colon is straightened. After surgery, fibrosis of the dense area between the anastomotic line and the sacrum occurs, and the rectum is fixed to the sacrum; these changes act in a similar fashion to sigmoid resection and proctopexy [15, 27]. Although our patient had a minimal distal margin of the rectum available for anastomosis, we determined that she was good candidate for sphincter preservation. We therefore performed Lap-LAR without rectopexy. At the time of writing this report, 12 months after surgery, our patient is doing well with no fecal incontinence or constipation. The outcome in our patient supports the use of this surgical procedure.

The prolapsing technique is useful for similar cases. In our patient, the tumor that filled the rectum was large and soft, and its cauliflower shape caused the friable edge to protrude laterally from its base, covering part of the rectal wall. These conditions posed two problems for conventional Lap-LAR: (1) although the distal margin of the rectum was minimally suitable for anastomosis, the dilated rectum hampered the laparoscopic view, compromising appropriate resection of the rectum; (2) the tumor rim would be included with the resected stump when the stapler made its cuts, possibly resulting in implantation of tumor cells. The prolapsing technique resolved these difficulties, enabling us to retract the tumor rim manually and to irrigate the rectum thoroughly. We were able to ensure the appropriate distal resection line, without including any part of the tumor with the cut edge of the rectum, under direct visualization. Intersphincteric resection of the rectum also may have enabled us to resect under direct visualization, but that technique is more invasive and increases the chance of seeding from the friable tumor. The use of Lap-LAR with the prolapsing technique is an established procedure, and our patient already had rectal prolapse, so the transanal eversion was easily accomplished [28, 29]. Our patient had good postoperative anal function and an adequate oncological resection margin. In our case, we did not create a temporary diverting stoma, because the anastomosis was performed without anxiety. However, Lap-LAR with the prolapsing technique sometimes requires a very low anastomosis, so a temporary diverting stoma should probably be used to protect an anastomosis. We speculate that the prolapsing technique not only satisfies the oncological view, but also preserves function and is a useful option for similar situations, such as a lower rectal cancer with a large, soft, friable tumor.

Etiologically, there is no apparent association between rectal cancer and rectal prolapse. Bowel intussusception in adults is typically due to a pathologic lead point (e.g., cancer, polyp) within the bowel that is pushed forward by normal peristalsis [30–32]. The same explanation may also fit rectal prolapse caused by rectal cancer. As mentioned above, there are three reported patients with RCRP, and all had relatively large tumors, with the rectum prolapsing and the tumor as a lead point. There are also case reports of large adenomas with rectal prolapse; in those patients, the tumor also functioned as a lead point [33, 34]. On the other hand, a previous retrospective study reported that patients with rectal prolapse have an increased risk for colorectal cancer (5.7 versus 1.4 percent in the control group; relative risk, 4.18; 95% confidence interval, 1.1–16.0) [35]. In any case, rectal prolapse is strongly related to colorectal cancer and we suggest that colorectal cancer should always be suspected when rectal prolapse is recognized.

## Conclusions

In conclusion, we performed Lap-LAR using the prolapsing technique for a patient with RCRP. Our case suggests that Lap-LAR can be an appropriate surgical procedure for this presentation and that rectopexy is not always necessary. The prolapsing technique is useful in selected patients.

## Abbreviations

Lap-LAR: Laparoscopic low anterior resection
RCRP: Rectal cancer with rectal prolapse
